# Supplementary material for: Turing patterns with high-resolution formed without chemical reaction in thin-film solution of organic semiconductors
Source: Nat Commun. 2022 Dec 2;13:7422. doi: 10.1038/s41467-022-35162-z (PMC9715637; doi:10.1038/s41467-022-35162-z)
Supplement: Supplementary file 1 — Supplementary Information [file 41467_2022_35162_MOESM1_ESM.pdf]

**Supplementary Information**

# Turing patterns with high-resolution formed without chemical reaction in thin-film solution of organic semiconductors

Xiang et al.

**Supplementary Note 1 | Estimation of trapped liquid film thickness between PDMS plate and substrate**

Silicon wafer and PDMS film (2-mm-thick) were treated with oxygen plasma for 3 min (Gas flow rate: 9.7 sccm; Pressure: 0.5 mbar; Power: 150 W). A drop of solution with controlled volume was casted onto silicon wafer with micropipette and subsequently pressed using the PDMS film, which was laminated on a glass-backplane, against the substrate with applied pressure of ~5 MPa. Then, we evaluated the area of the spread liquid film sandwiched between the substrate and PDMS. The liquid film thickness was estimated with dividing the liquid volume by the estimated area of the liquid film. The estimated liquid thickness is 400~600 nm.

**Supplementary Note 2 | Self-catalysis and concentration-wave formation in an uphill-diffusion system**

In a molecule-solvent binary spinodal system (M-S), when the system is driven into a state of oversaturation (through solvent evaporation or cooling the solution from high temperature), the system becomes unstable. We use one-dimensional model to illustrate the wave formation process. If there is a small undulation of concentration at a point (small concentration enhancement of M, indicated by 1), a concentration gradient at the left and right side of the small hump is created (around point a) (Supplementary Fig. 4a). Because of the uphill diffusion, the solute M around the hump will diffuse to the hump region, and the hump is enlarged gradually. This leads a depletion of M at the two sides of the hump and this is expressed with two negative humps (indicated by 2).

Again, new regions of concentration gradient are created (indicated by point b) (Supplementary Fig. 4b). The uphill-diffusion of M creates two new humps (indicated by 3) (Supplementary Fig. 4c). This process further continues (Supplementary Fig. 4d) and forms a concentration wave. We would like to stress that the solvent diffusion is not shown in the figure for simplifying the illustration. The diffusion of solvent is in the opposite direction of solute molecule diffusion during the formation of concentration waves.

**Supplementary Note 3 | Undercooling and energy stored in solution film**

We propose a simple expression of energy stored in undercooled solution. A schematic illustration of the molecules-solvent binary spinodal system is shown in Supplementary Fig. 5. For a state (concentration $C$, temperature $T$) within the spinodal region, where the homogeneous solution is unstable against infinitesimal fluctuations in density or concentration and separates into two phases with well-defined size, i.e., spinodal precipitation (inset). We examine a solution with concentration C. When the solution is at point P which is located above the point Q on the spinodal curve, the solution is clear. If the solution is gradually cooled down to room temperature $T_r$, a spinodal precipitation occurs and a random structure will be obtained. Assume that one can maintain the system very stable during the solution cooling to temperature $T_r$ without the happening of precipitation. Then, the solution is oversaturated, and the degree of oversaturation can be expressed with undercooling $\Delta T=T_{sp}-T_r$. In our system, the experiment starts from the state ($C_0$, $T_r$) with $T_r$ keeps unchanged. With solvent evaporation, the

concentration moves through C′ to concentration C without precipitation. In this case, the solution is also under saturated condition and can be expressed with undercooling (i.e., $\Delta T$) which is induced by concentration change. Such concentration-induced undercooling is named as concentration undercooling in solidification physics. We can approximately express the energy of the undercooled liquid film with undercooling-induced energy-deviation from equilibrium (with reference to the equilibrium phase, i.e., transition point at spinodal curve) and surface energy. For a unit volume of solution, the former can be expressed as $\Delta T \times C_p$ and the latter can be expressed as $(\sigma_1+\sigma_2)\times S_{int}$. where $C_p$ is specific heat, $\sigma_1$ and $\sigma_2$ is the energy density of interfaces between PDMS/solution and solution/substrate, $S_{int}$ is the area of single interface for unit volume solution. A unit volume of solution is expressed as $1=(a+b)\ S_{int}$, where $a+b$ is the thickness of solution. Thus, $S_{int}=1/(a+b)$ and we have:

$$E= \Delta T \times C_p + (\sigma_1+\sigma_2)/(a+b) \quad (1)$$

where $\Delta T$ can be expressed by $\beta(C-C')$, and $C'$ is concentration on the spinodal curve corresponding to experimental temperature, $\beta$ is the slope of left branch of the spinodal cure. Then we have:

$$E = \beta(C - C')C_p + (\sigma_1 + \sigma_2)/(a + b) \quad (2)$$

This is a very rough expression for the energy ~ concentration dependence. Better expression which contains more information is required.

## Supplementary Note 4 | Observation of line instability

We have attempted to understand the line instability and branching mechanism use in-

situ optical microscopic observation. As the allowed observation time and field of view is very limited, we use 1,2-dichlorobenzine as solvent (10 mg C8-BTBT/ml) to extent the drying time. Although the formed structures are much less regular than that formed with C8-BTBT-chlorobenzene system, it still allows us to capture many useful information. Supplementary Fig. 8 shows movie frames of different moments taken during the experiment. Frame (a) in Supplementary Fig. 8 shows no clear sign of pattern formation. In frame (b) of Supplementary Fig. 8 we see an emerged line (we name it as first primary line) and branching from it; while second primary line generation starts to begin. In frame (c) of Supplementary Fig. 8, the emerged second primary line repeats the branching process of the first primary line, and third primary line generation starts to begin. One can see that the branches grown from the first primary line and second primary line are nearly collectively aligned. Such sequentially repeated line-generation and line-branching is the mechanism of forming 2D patterned structure through line instability.

**Supplementary Note 5 | Ternary phase diagram and effect of introduction of PS**

Through literature search we can sketch a schematic ternary diagram at experimental temperature as shown in the Supplementary Fig. 9 [1]. The system contains two organic solutes and one solvent (for example: small molecule-polymer-solvent, or polymer-polymer-solvent). We use small molecule and polymer instead of the C8-BTBT and PS in the diagram, and take two points "D" and "E" to describe the effect of polymer addition. The three components of a point in the diagram can be read out from three

component-axes of the diagram (sides of triangle). For instance, one can read out the three components of the point E (small molecule, polymer, solvent) with the length scale of $\overline{CQ}$, $\overline{BP}$, and $\overline{AM}$. We inspect two samples made with prepared solutions at points D and E (hereafter we name the two samples as sample D and sample E, respectively). The two samples contain the same proportion of solvent, and also the same proportion of solute (small molecule + polymer), because both points locate on the line MN. The polymer concentration of the sample E is higher than the sample D. During experiment, with solvent evaporation the ratio of the small-molecule/polymer will not change in the samples before precipitation happens. For the sample D, the concentration will move along the line DF with solvent evaporation. While, for the sample E, the concentration will move along the line EG. We expect that the sample E will reach spinodal curve faster than sample D, as the solvent contained in point G is more than that in the point F. Therefore, a larger undercooling is expected for sample E when spinodal precipitation occurs.

**Supplementary Note 6 | Patterning of P3HT/perylene and AFM/X-ray analysis**

Solutions of poly(3-hexylthiophene) (P3HT, regioregular) and perylene were prepared with concentration 10mg/ml in chlorobenzene. The pattern generation process is identical to that for C8-BTBT patterning. For the P3HT, array of lines is preferred pattern generated and the pattern morphology is sensitive to small variation of experimental condition. Modified line patterns, like modulated lines or mosaic-like patterns can be observed. No crystal grains can be observed with AFM investigation

because the P3HT is a polymer material (Supplementary Fig.10a and Fig.10b). The nanometer-sized feature in the Supplementary Fig.10b might be related with polymer chain staking or folding. X-ray analysis shows that the material is mainly amorphous with a little sign of crystallization which is probably caused by ordering or folding of P3HT chains (Supplementary Fig.11a) [2]. This agrees with the observed nanofeature in Supplementary Fig.10b. For the perylene, line patterns were found. Both AFM and X-ray analysis showed strong crystalline phase (Supplementary Fig.10c, Fig.10d and Fig.11b). Although both C8-BTBT and perylene are small molecule semiconductor materials, the patterned perylene shows strong crystal morphology. This might be originated from different mechanism of crystallization of the two materials. Crystallization is controlled by nucleation and growth process. For the C8-BTBT, the crystallization is probably mainly controlled by nucleation process (number of nuclei is large, the growth of crystal is slow). Therefore, solvent vapor annealing (SVA) is often used to generate proper sized crystals after film deposition [3]. For the perylene, the crystallization process is mainly controlled by growth process, and large sized crystals can be prepared by simple solution drop-casting [4]. Although patterned perylene crystal can be fabricated, the charge mobility of perylene crystal is low [4,5]. Therefore, finding organic semiconductor materials to generate patterns of single crystals with high charge mobility is interesting for device fabrication.

## Supplementary Note 7 | Experiment for exclusion of Rayleigh-Bénard (R-B) mechanism

Rayleigh-Bénard system is formed with two parallel plates, and a liquid film is sandwiched between them. Rayleigh-Bénard pattern-generation requires a balance between upwelling induced by temperature gradient and downward flow caused by gravity. In this case, the temperature of bottom plate is higher than that of the top plate, i.e., the direction of gravity (g) is the same as the direction of temperature gradient $\nabla T$ (the temperature gradient is directed from low temperature to high temperature). In our experiment, if the patterns are generated by Rayleigh-Bénard mechanism the temperature of bottom plate must be higher than the top plate for some reason (Supplementary Fig. 12a). Once we turn the system upside down, the direction of gravity will not consistent with the direction of temperature gradient (Supplementary Fig. 12b). This means that if we can observe Rayleigh-Bénard patterns for the first case they will not exist in the second case.

**Supplementary Note 8 | Mechanism of pattern condensation from solutions onto the substrates**

The pattern condensation onto a substrate could be completed with following proposed process (Supplementary Fig. 13). With the concentration-wave generation, precipitation starts from the peak regions of the waves and deposits onto the substrate to from a solid (or half-solid) pattern. At this stage the solution maintains a continuous liquid film. At later stage, with further reduction of the liquid volume, massive air is trapped-in and free liquid surface forms. Since the substrate surface is more hydrophilic the residual liquid prefers to stay at the substrate side. The organic materials are

hydrophobic (see Supplementary Fig. 14) and the residual liquid is patterned into small units by the structured organic materials. The perimeters of the patterned liquid are pinned at the edges of the organic structures. This proposal is supported by previous works, where surface energy barrier created with organic molecules has been used to pattern liquid [6]. The surface tension of the patterned liquid induces a coffee-stain effect where the solute is driven further to the edge regions until the residual liquid dries out [7]. The proposed process is also supported with some defects of the generated patterns where the coffee-stain effect did not work effectively, and the patterned small liquid dried out uniformly (Supplementary Fig. 15).

**Supplementary Note 9 | Solvent evaporation and solution concentrating process**

During solvent evaporation the solvent molecules leave from the edges of sample to open air (see Supplementary Fig.16a). Let's consider the situation where the mobility of solvent molecules (blue circles) is much larger than that of the solute molecules (black circles) which is reasonable for most cases, because the smaller mass of solvent molecule and interaction between the substrate and solute molecules [8]. The solvent evaporation starts from sample edges (I, II in Supplementary Fig.16a). The top pressure application and capillary effect will force the liquid to fill up the space between the top-plate and bottom-plate. When a solvent molecule leaves from the liquid, a vacancy space is created at the edge of liquid film and it diffuses inward (II, III in Supplementary Fig.16a). The movement of vacancy inward equivalents to a movement of solvent molecule outward. The process continues (IV, V in Supplementary Fig.16a) and more

molecules leave from the sample, and equivalently more solvent molecules move outwards to supplement the vacancy spaces. The vacancies transfer inward and eventually vanish at the liquid/plate interfaces, and the thickness of the liquid film is reduced (VI in Supplementary Fig.16a). In this case, the solute concentration in the film is uniformly distributed before precipitation, because the much faster movement of solvent molecules than the solute molecules. In other words, the increased solute concentration at marginal area caused by solvent evaporation can be diluted quickly by the supplied solvent from the internal area. We would like to stress that the molecular movement is not purely controlled by normal diffusion, liquid rheology will play certain role due to the pressure application and capillary force. The rheology does not affect our qualitative discussion, since the evaporation is a slow and steady process in our experiments. The rheology can result an increased unidirectional mobility of solvent molecules (outward). If the mobility of the solvent molecules is not large enough in comparison with solute molecule, a concentration gradient of solute may build up. This may not greatly affect the pattern formation process, because the pattern formation can occur in a wide range of solute concentrations as shown in our experiments. Supplementary Fig.16b is schematic drawing for analysis, where the red line represents a simplified concentration distribution C(x), and $\Delta C(x)$ is the concentration deviation in comparison with the concentration in the center area of sample $C_C$. While, the black curve represents the spinodal wave if the instability occurs in the uniform solution film with concentration $C_C$. If the concentration deviation $\Delta C(x)$ does not involve the spinodal precipitation, the resulting concentration wave will be shown as the blue curve

in Supplementary Fig.16c which is the superposition of the black curve and ΔC(x) in Supplementary Fig.16b. In reality, the ΔC(x) is a part of solution concentration C(x) and it will involve the spinodal precipitation. This leads an enlargement of the amplitude of the blue wavy curve, i.e., the red curve in Supplementary Fig.16c. Therefore, even at the situation of existing a moderate concentration gradient caused by solvent evaporation the spinodal precipitation can still be observed.

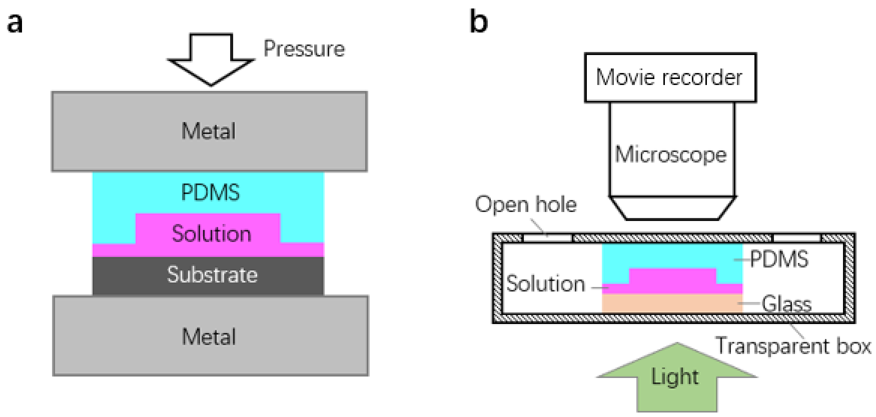


**Supplementary Fig. 1 |** Schematic illustrations of experimental setup used in this work for (a) ex-situ and (b) in-situ observation.

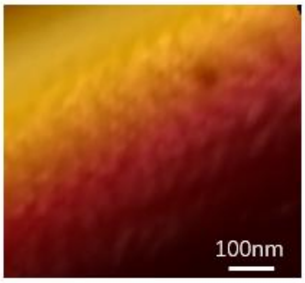


**Supplementary Fig. 2 |** AFM image of a patterned C8-BTBT+PS (1:1) line which shows nanocrystalline structure of C8-BTBT.

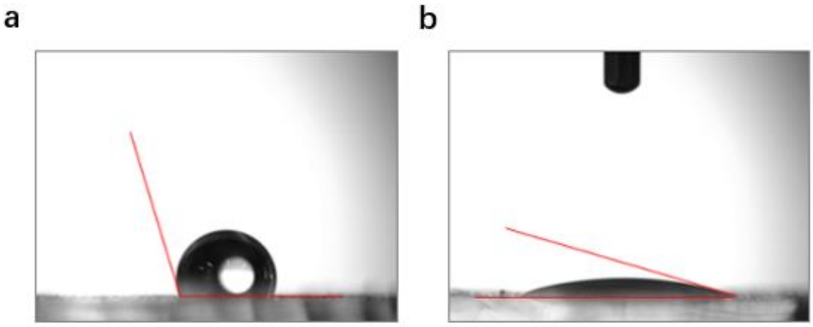


**Supplementary Fig. 3 |** Contact angle measurement of water droplets on surfaces of PDMS plate. (a) Contact angle measured before plasma treatment (109.96°). (b) Contact angle measured after treatment with oxygen plasma for 3 min (10.44°).

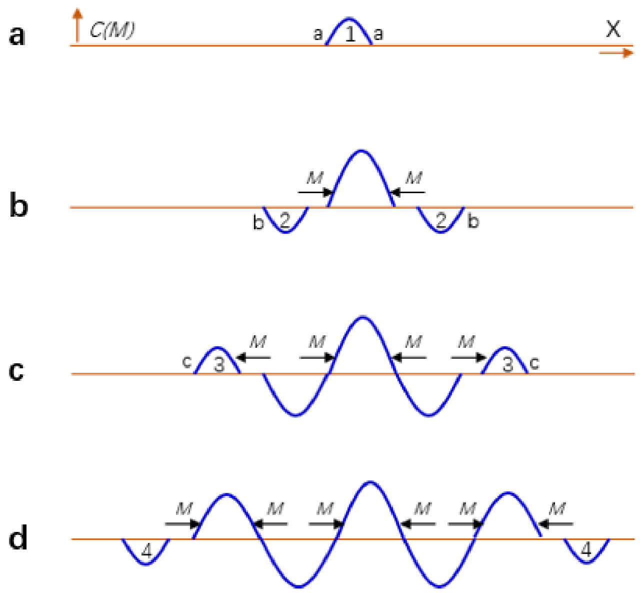


**Supplementary Fig. 4 |** Schematic illustration of concentration-wave formation process in an uphill-diffusion driven binary spinodal solution system. (a) Small undulation of concentration (hump 1) is created. (b) The hump grows gradually and depletion of solute at the two sides of the hump is created which is shown as negative humps (humps 2). (c) The two negative humps grow and positive humps are developed next to them (humps 3). (d) The positive humps grow and more negative humps (humps 4) are created next to them. The system is a solution formed with molecules (M) and solvent (S). For simplicity, only molecule diffusion direction is indicated (black arrows). The molecule concentration $C(M)$ and position (X) are indicated in (a).

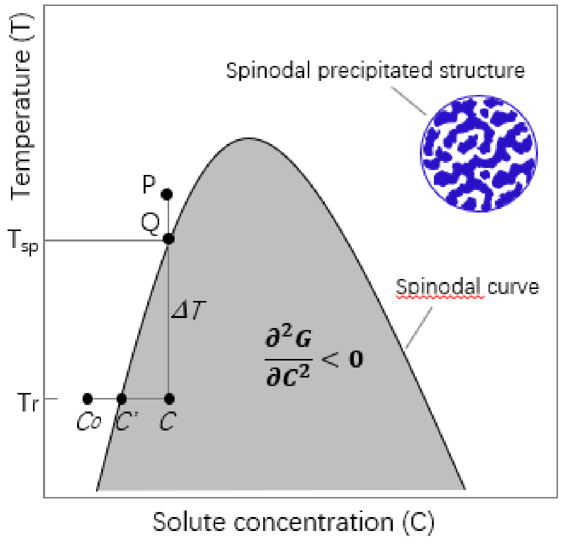


**Supplementary Fig. 5 |** Schematic phase diagram of a molecule-solvent binary system. Inset shows random spinodal structure formed under equilibrium.

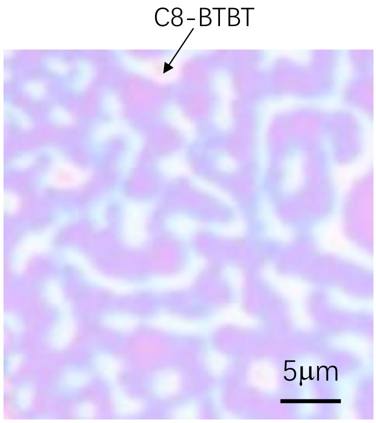


**Supplementary Fig. 6 |** Typical spinodal structure observed on casted C8-BTBT thin film (on $SiO_2$/Si substrate) with solution concentration of 5 mg/ml in chlorobenzene.

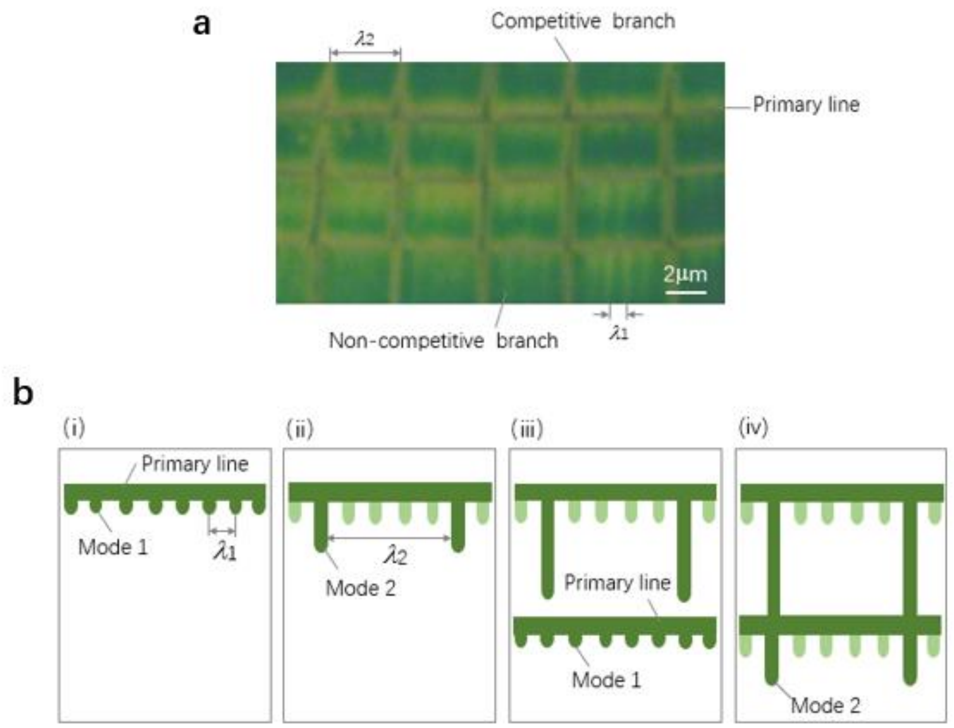


**Supplementary Fig. 7 |** Optical image of an underdeveloped square-grid pattern and proposed pattern formation mechanism. (a) Optical image of a square-grid pattern during development. (b) Proposed pattern formation process. The pattern formation starts from instability of primary line (i), and this initial instability is governed by mode-1 with period $\lambda_1$. However, this mode may not be a competitive mode (ii), and only one mode became dominant eventually (indicated as "competitive branches" in (a), mode-2 with period $\lambda_2$). The next primary line is emerged after the branching of the primary line emerged before it, and it will repeat the branching process (iii) (iv). All other non-competitive modes will disappear eventually. If the initial instability with short wavelength can grow up and the competition from the long wavelength mode can be avoided, a fence-like pattern will form.

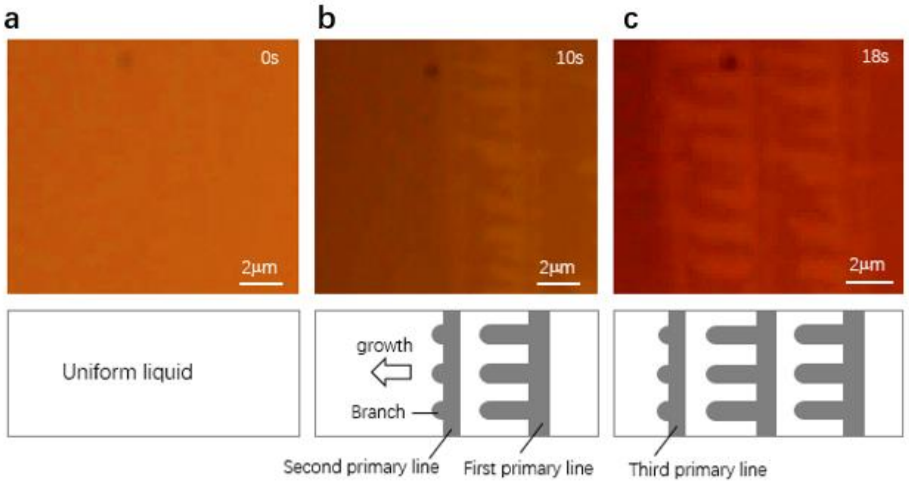


**Supplementary Fig. 8 |** Movie frames of different moments taken during pattern formation experiment with C8-BTBT solution of 1,2-dichlorobenzine. (a) Uniform liquid film before pattern formation. (b) Emerged primary line and branching from it, and second primary line generation starts subsequently. (c) The emerged second primary line repeats the branching process of the first primary line, and third primary line generation starts to begin. The branches grown from the first primary line and second primary line are nearly collectively aligned. The corresponding bottom panel of each movie frame illustrates the structure formation process. The start of time counting (t=0) is arbitrary chosen from the movie.

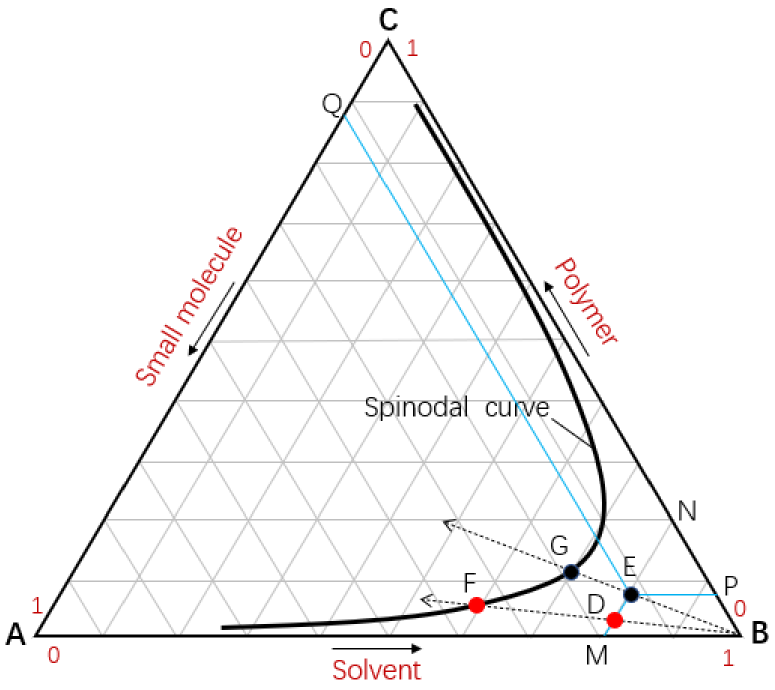


**Supplementary Fig. 9 |** Schematic illustration of ternary phase diagram of small molecule-polymer-solvent system.

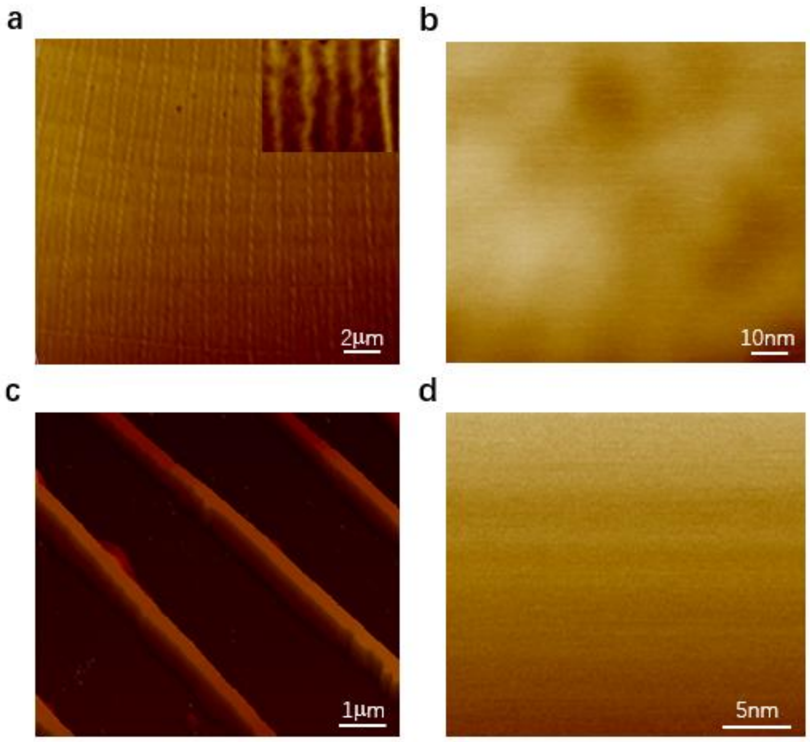


**Supplementary Fig. 10 |** AFM images of patterned P3HT and Perylene with different magnifications. (a) Image of patterned P3HT and (b) zoom-in image of local area with a line. (c) Patterned perylene lines and (d) zoom-in image of local area on the top of a line.

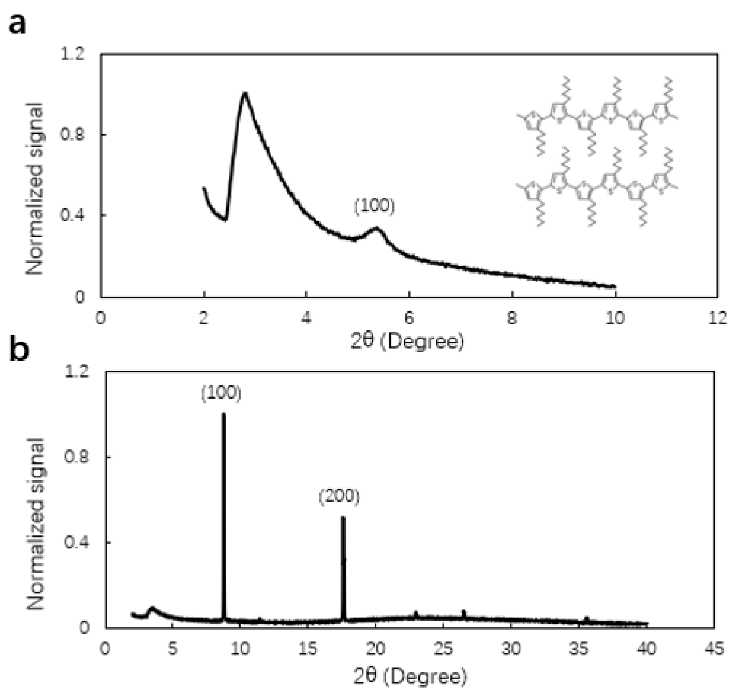


**Supplementary Fig. 11 |** Results of X-ray analysis for patterned materials. (a) X-ray diffraction spectrum taken from patterned P3HT. The inset shows a schematic illustration of chain stacking of P3HT. (b) Diffraction spectrum taken from patterned perylene.

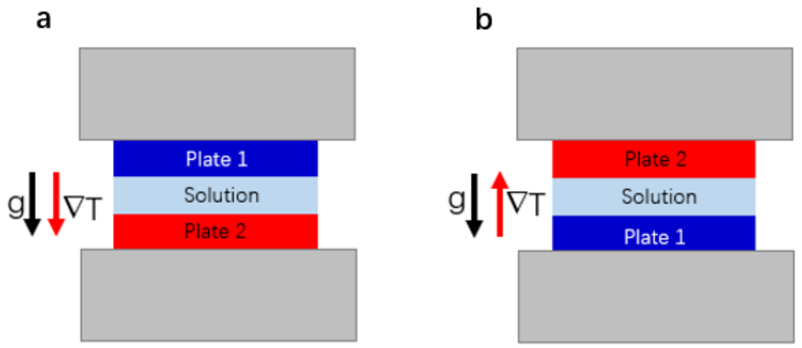


**Supplementary Fig. 12 |** Schematic illustrations of experimental systems for exclusion

of Rayleigh-Bénard (R-B) mechanism. (a) Experimental system with normal arrangement. (b) Experimental system with upside-down arrangement. If R-B pattern is observed in the case (a), the gravity ‘g’ and temperature gradient ‘∇T’ must point in the same direction (down). Then, in the case (b) the R-B pattern will not be observed, since the gravity ‘g’ and temperature gradient ‘∇T’ no longer point in the same direction.

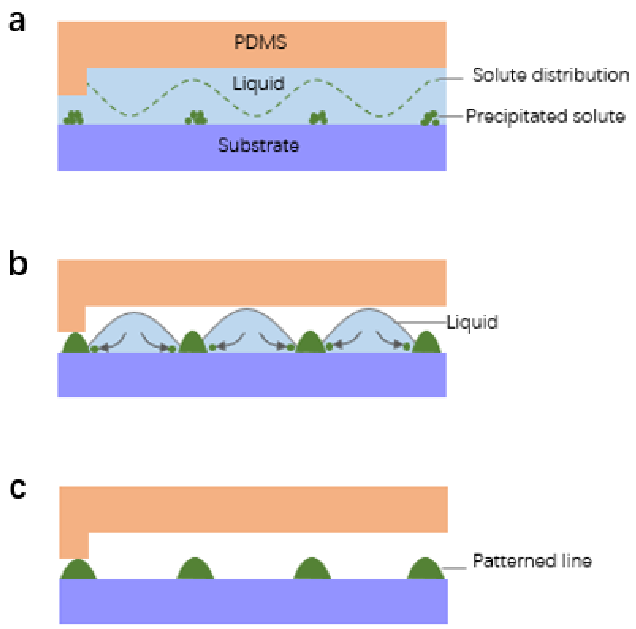


**Supplementary Fig. 13 |** Proposed pattern deposition process from liquid to substrate. (a) Precipitation starts from the peak regions of the waves and the solute is deposited onto the substrate. (b) At a later stage, with reduction of liquid, the air starts to trap-in, and free liquid surface forms. The residual liquid film is patterned into small units whose edges are pinned at the precipitated solute lines. The surface tension of the small liquid induces a coffee-stain effect where the solute is driven further to the edge regions. (c) Patterned material is formed after drying out of liquid.

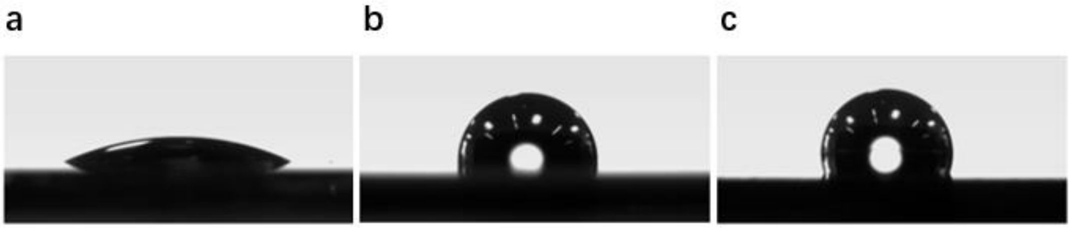


**Supplementary Fig. 14 |** Contact angle measurements of water droplets on different film surfaces. (a) Pure C8-BTBT. (b) Pure PS. (c) C8-BTBT + PS (1:1).

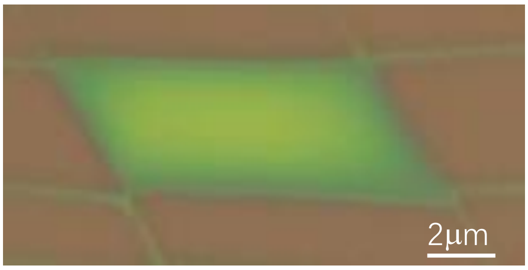


**Supplementary Fig. 15 |** A square shaped C8-BTBT film framed with generated lines. The thin film was dried from a small liquid with 4-side edges pined at the surrounding lines.

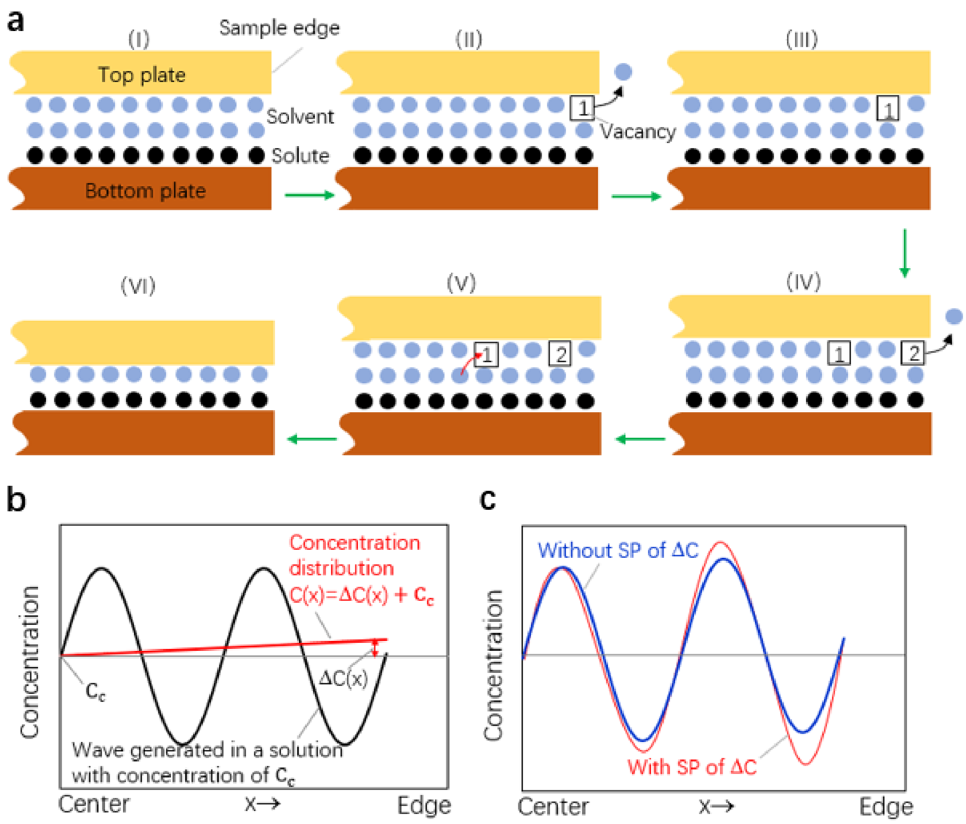


**Supplementary Fig. 16 |** Schematic sketch to illustrate the solvent evaporation and solution concentrating process. (a) Schematic illustration of solvent evaporation and solution concentrating process, where a much higher mobility of solvent molecules in comparison with solute molecules is assumed. For a clear illustration the solvent molecules (blue circles) and the solute molecules (black circles) were drawn in different layers. (I) Thin solution film sandwiched between two plates; (II) A solvent molecule leaves from sample edge and a vacancy (numbered with 1) is created; (III) Vacancy 1 diffuses inward through replacement with a solvent molecule; (IV) Vacancy 1 moves further inward and next vacancy (numbered with 2) is created by solvent evaporation;

(V) Vacancies move further inwards. Red arrow shows that a solvent molecule can move into a vacancy from various directions; (VI) Reduction of liquid film thickness. (b) Schematic illustration of concentration deviation from sample center to edge (red line) and concentration wave in a solution film with average concentration $C_C$. (c) Schematic illustration of concentration waves when ΔC(x) does not involve spinodal precipitation (SP) (blue curve) and ΔC(x) involves the spinodal precipitation (red curve).

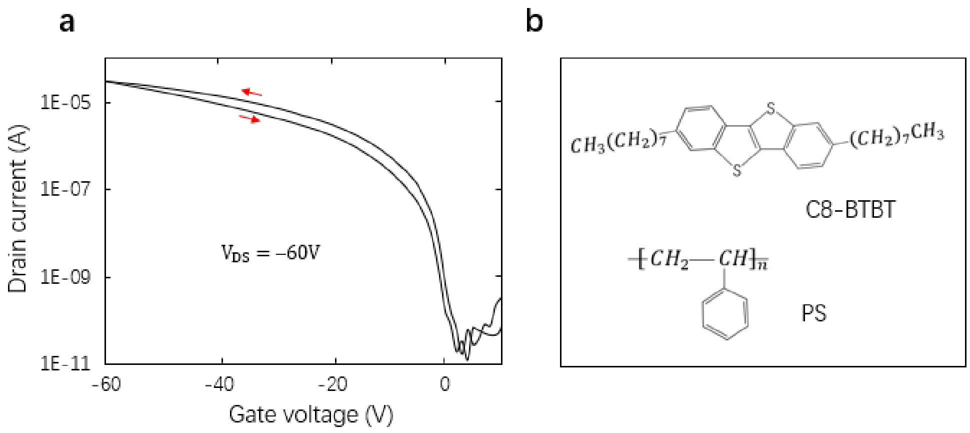


**Supplementary Fig. 17 |** Hysteresis of transfer characteristic curves and molecule structures of materials used for the device. (a) Transfer characteristic curves recorded with forward/reverse gate-voltage scans. The curves were taken from the same device as shown in Fig.4e and Fig.4f. (b) Molecule structures of C8-BTBT and PS.

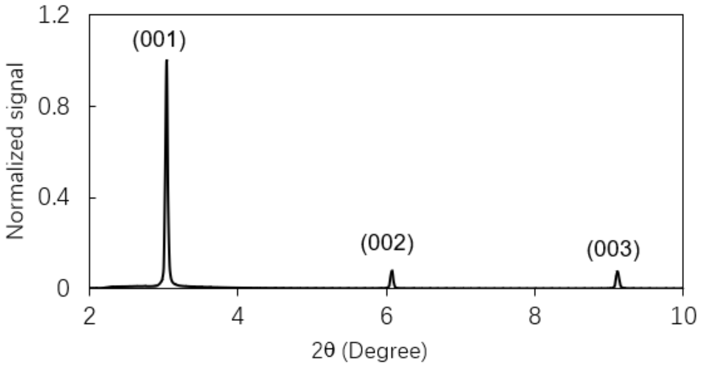


**Supplementary Fig. 18 |** X-ray diffraction spectrum taken from patterned C8-BTBT structure without annealing.

**Supplementary Table 1 |** Examples of patterning organic semiconductor (OSC) with stamping techniques.

| No | Schematic illustrations | Brief description | Ref |
|---|---|---|---|
| 1 | 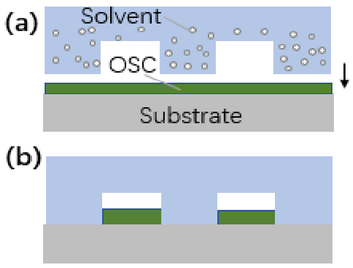  | Patterned PDMS stamp is dipped into solvent to absorb the solvent molecules. Subsequently, the PDMS stamp is firmly contacted with an organic semiconductor (OSC) film. The film is selectively dissolved (with the solvent diffused out) and patterned. | 9 |
| 2 | 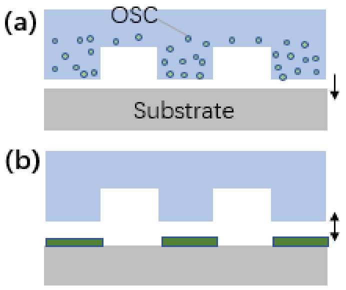  | Patterned PDMS stamp is dipped into OSC solution and dried afterwards. Then, the PDMS stamp is contacted onto a substrate surface and the OSC molecules are transferred to the substrate surface via out-diffusion. | 10 |
| 3 | 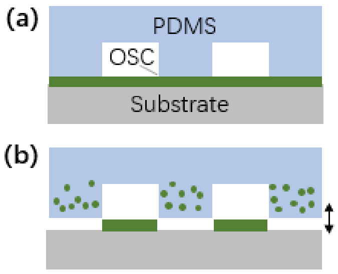  | Use a PDMS stamp to selectively remove OSC film deposited on a substrate. The PDMS stamp is firmly contacted with the precoated OSC film and the OSC molecules diffuse into the PDMS material. | 11-13 |
| 4 | 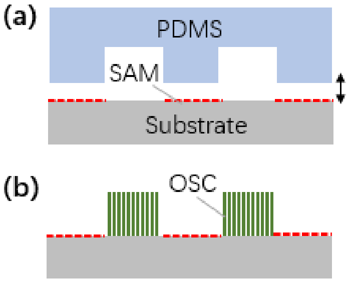  | Selective growth of vertical nanowires of OSC on patterned substrate made with stamping technique. The fabrication principle used is that the nanowires, grown by physical vapor deposition, tend to grow within the hydrophilic areas. | 14 |
| 5 | 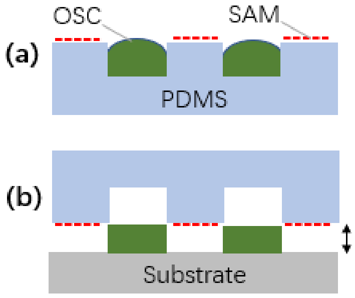  | Micro/nano molding. Micro/nano structured material is produced by selectively inking a PDMS stamp with OCS solution. Then, the structured OCS templated by the PDMS is transferred to a substrate surface. | 15 |

| | | | |
|---|---|---|---|
| 6 | 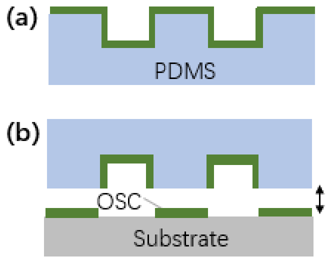 | Material transfer from PDMS stamp to substrate. OSC film is formed on the structured surface of a PDMS stamp by spin-coating or other deposition techniques. Then, the OSC is selectively transferred onto a substrate surface. | 16 |
| 7 | 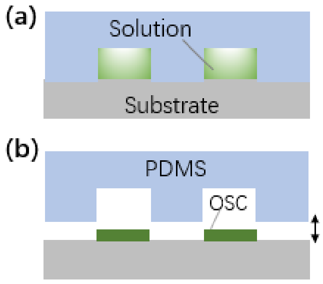 | Solution is dispensed onto a substrate and a patterned PDMS is brought to contact with the inked substrate. The solution is segregated into the grooves of the stamp through capillary effect and dried there. | 17,18 |
| 8 | 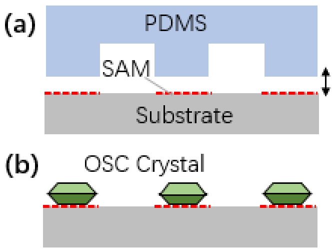 | Vapor growth of OSC single crystal arrays on a patterned substrate through controlled nucleation. The nucleation sites were defined by soft contact printing with a PDMS stamp. | 19 |
| 9 | 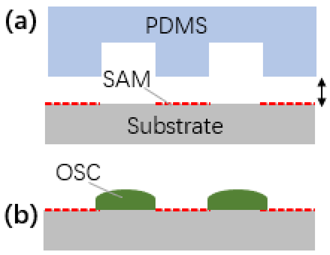 | Use a PDMS stamp to print surface energy pattern on a substrate, and the deposited OSC solution is patterned with the wetting contrast on the substrate and selectively dried on the hydrophilic areas. | 20 |
| 10 | 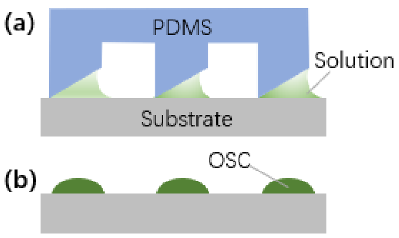 | Fabrication of OSC arrays by a stamp with an inclined slope structure. Solution under such a stamp is distributed under the inclined features caused by capillary effect during drying. | 21,22 |

| | | | |
|---|---|---|---|
| 11 | 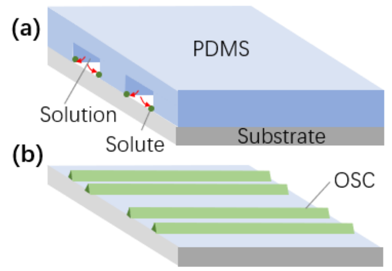 | Stamp guided side deposition. Drying solution confined in channels of structured PDMS stamp induces a convection which transfer OSC molecules to the channel edges. Submicron sized OSC wires can be produced. | 23,24 |
| 12 | 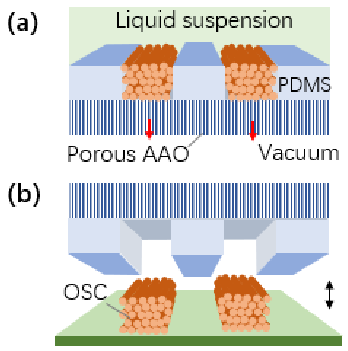 | Dispersed OSC wires in liquid suspension are deposited into the trenches of a stamp forced by solution outflow through porous materials induced by a pressure difference. Then, the aligned patterned wires are transferred onto substrate surface. | 25 |
| 13 | 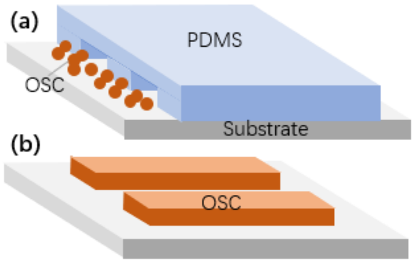 | An OSC material with low melting point is placed at the entrance of channels of a PDMS stamp. Heating the OSC to a temperature above its melting point and the channels are filled with capillary action. | 26 |
| 14 | 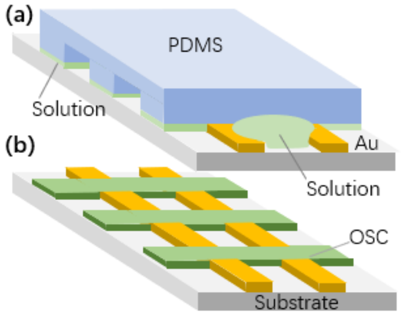 | A structured stamp is placed on pre-patterned electrode stripes. Solution dispensed at the edge of the stamp is sucked into underneath space of the stamp with capillary force and dried there. | 27,28 |
| 15 | 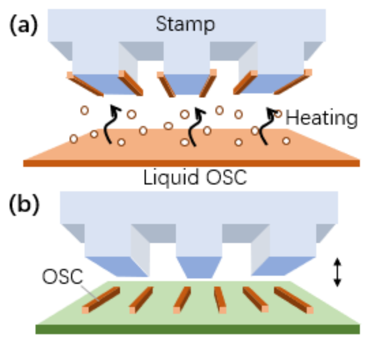 | Stamp is used to guide the crystal nucleation and growth of OSC materials. OSC vapor is deposited and crystalized on the edges of pillar structure with the help of wetting contrast between top and vertical areas induced by surface roughness change. The OSC pattern can be transferred to a substrate. | 29 |

## Supplementary References


1. Schaefer, C., Michels, J. J., van der Schoot, P. Structuring of thin-film polymer mixturesupon solvent evaporation. *Macromolecules* **49**, 6858-6870 (2016).
2. Zhokhavets, U., Erb, T., Hoppe, H., Gobsch, G., Serdar Sariciftci, N. Effect of annealing of poly(3-hexylthiophene)/fullerene bulk heterojunction composites on structural and optical properties. *Thin Solid Films* **496**, 679-682 (2006).
3. Liu, C., Minari, T., Lu, X., Kumatani, A., Takimiya, K., Tsukagoshi, K. Solution-processable organic single crystals with bandlike transport in field-effect transistors. *Adv. Mater.* **23**, 523–526 (2011).
4. Liao, Q., Zhang, H., Zhu, W., Hua, K., Fu, H. Perylene crystals: tuning optoelectronic properties by dimensional-controlled synthesis. *J. Mater. Chem. C* **2**, 9695 (2014).
5. Lee, J-W., Kang, H-S., Kim, M-K., Kim, K., Cho, M-Y., Kwon, Y-W., Joo, J. Electrical characteristics of organic perylene single-crystal-based field-effect transistors. *J. Appl. Phys.* **102**, 124104 (2007).
6. Wang, J. Z., Zheng, Z. H., Li, H. W., Huck, W. T. S., Sirringhaus, H. Dewetting of conducting polymer inkjet droplets on patterned surfaces. *Nat. Mater.* **3**, 171-176 (2004).
7. Deegan, R. D., Bakajin, O., Dupont, T. F., Huber, G., Nagel, S. R., Witten, T. A. Capillary flow as the cause of ring stains from dried liquid drops. *Nature* **389**, 827-829 (1997).
8. Shang, Y., Kazmer, D., Wei, M., Barry, C., Mead, J. Numerical simulation of the self-assembly of a polymer–polymer–solvent ternary system on a heterogeneously functionalized substrate. *Polym. Eng. Sci.* **50**, 2329-2339 (2010).
9. Kim, K., Jang, M., Lee, M., An, T. K., Anthony, J. E., Kim, S. H., Yang, H., Park, C. E. Unified film patterning and annealing of an organic semiconductor with micro-grooved wet stamps. *J. Mater. Chem. C* **4**, 6996-7003 (2016).
10. Lee, K., Kim, J., Shin, K., Kim, Y. S. Micropatterned crystalline organic semiconductors via direct pattern transfer printing with PDMS stamp. *J. Mater. Chem.* **22**, 22763 (2012).
11. Dickey, K. C., Subramanian, S., Anthony, J. E., Han, L., Chen, S., Loo, Y. Large-area patterning of a solution-processable organic semiconductor to reduce parasitic leakage

and off currents in thin-film transistors. *Appl. Phys. Lett.* **90**, 244103 (2007).

12. Park, H-L., Lee, B-Y., Kim, S-U., Suh, J-H., Kim, M-H., Lee, S-D. Importance of surface modification of a microcontact stamp for pattern fidelity of soluble organic semiconductors. *J. Micro/Nanolith. MEMS MOEMS* **15**, 013501 (2016).
13. Bae, I., Kang, S. J., Shin, Y. J., Park, Y. J., Kim, R. H., Mathevet, F., Park, C. Tailored single crystals of triisopropylsilylethynyl pentacene by selective contact evaporation printing. *Adv. Mater.* **23**, 3398–3402 (2011).
14. Zhao, Y. S., Zhan, P., Kim, J., Sun, C., Huang, J. Patterned growth of vertically aligned organic nanowire waveguide arrays. *ACS Nano* **3**, 1630-1636 (2010).
15. Park, K. S., Cho, B., Baek, J., Hwang, J. K., Lee, H., Sung, M. M. Single-crystal organic nanowire electronics by direct printing from molecular solutions. *Adv. Funct. Mater.* **23**, 4776–4784 (2013).
16. Takakuwa, A., Azumi, R. Influence of solvents in micropatterning of semiconductors by microcontact printing and application to thin-film transistor devices. *Jpn. J. Appl. Phys.* **47**, 1115-1118 (2008).
17. Jo, P. S., Vailionis, A., Park, Y. M., Salleo, A. Scalable fabrication of strongly textured organic semiconductor micropatterns by capillary force lithography. *Adv. Mater.* **24**, 3269–3274 (2012).
18. Watanabe, S., Fujita, T., Ribierre, J., Takaishi, K., Muto, T., Adachi, C., Uchiyama, M., Aoyama, T., Matsumoto, M. Microcrystallization of a solution-processable organic semiconductor in capillaries for high-performance ambipolar field-effect transistors. *ACS Appl Mater Interfaces* **8**, 17574-17582 (2016).
19. Briseno, A. L., Mannsfeld, S. C. B., Ling, M. M., Liu, S., Tseng, R. J., Reese, C., Roberts, M. E., Yang, Y., Wudl, F., Bao, Z. Patterning organic single-crystal transistor arrays. *Nature* **444**, 913-917 (2006).
20. Briseno, A. L., Roberts, M., Ling, M. M., Moon, H., Nemanick, E. J., Bao, Z. Patterning organic semiconductors using "dry" poly(dimethylsiloxane) elastomeric stamps for thin film transistors. *J. Am. Chem. Soc.* **128**, 3880-3881(2006).
21. Nakayama, K., Hirose, Y., Soeda, J., Yoshizumi, M., Uemura, T., Uno, M., Li, W., Kang, M. J., Yamagishi, M., Okada, Y., Miyazaki, E., Nakazawa, Y., Nakao, A., Takimiya, K., Takeya, J. Patternable solution-crystallized organic transistors with high charge carrier mobility. *Adv. Mater.* **23**, 1626–1629 (2011).
22. Diao, Y., Shaw, L., Bao, Z., Mannsfeld, S. C. B. Morphology control strategies for solution processed organic semiconductor thin films. *Energy Environ. Sci.* **7**, 2145–2159 (2014).
23. Li, J., Chang, X., Li, S., Shrestha, P. K., Tan, E. K. W., Chu, D. High-resolution electro-chemical transistors defined by mould-guided drying of PEDOT:PSS liquid suspension. *ACS Appl. Electron. Mater.* **2**, 2611(2020).

24. Li, S., Chun, Y. T., Zhao, S., Ahn, H., Ahn, D., Sohn, J. I., Xu, Y., Shrestha, P., Pivnenko, M., Chu, D. High-resolution patterning of solution-processable materials via externally engineered pinning of capillary bridges. *Nat. Commun*. **9**, 393 (2018).
25. Oh, J. H., Lee, H. W., Mannsfeld, S., Stoltenberg, R. M., Jung, E., Jin, Y. W., Kim, J. M., Yoo, J. B., Bao, Z. Solution-processed, high-performance n-channel organic microwire transistors. *PNAS* **106**, 6065-6070 (2009).
26. Kim, A., Jang, K-S., Kim, J., Won, J. C., Yi, M. H., Kim, H., Yoon, D. K., Shin, T. J., Lee, M-H., Ka, J-W., Kim, Y. H. Solvent-free directed patterning of a highly ordered liquid crystalline organic semiconductor via template assisted self-assembly for organic transistors. *Adv. Mater.* **25**, 6219–6225 (2013).
27. Cavallini, M., D'Angelo, P., Criado, V. V., Gentili, D., Shehu, A., Leonardi, F., Milita, S., Liscio, F., Biscarini, F. Ambipolar multi-stripe organic field-effect transistors. *Adv. Mater.* **23**, 5091–5097 (2011).
28. Zhang, X. J., Jie, J., Deng, W., Shang, Q., Wang, J., Wang, H., Chen, X., Zhang, X. H. Alignment and patterning of ordered small-molecule organic semiconductor micro-/nanocrystals for device applications. *Adv. Mater.* **28**, 2475–2503 (2016).
29. Wu, Y., Feng, J., Jiang, X., Zhang, Z., Wang, X., Su, B., Jiang, L. Positioning and joining of organic single-crystalline wires. *Nat. Commun*. **6**, 6737 (2015).
