# Supplementary material for: Turing patterns with high-resolution formed without chemical reaction in thin-film solution of organic semiconductors
Source: Nat Commun. 2022 Dec 2;13:7422. doi: 10.1038/s41467-022-35162-z (PMC9715637; doi:10.1038/s41467-022-35162-z)
Supplement: Supplementary file 3 — Description of Additional Supplementary Files [file 41467_2022_35162_MOESM3_ESM.pdf]

File name: Supplementary Movie 1

Description: The process of pattern formation in solution film.

File name: Supplementary Movie 2

Description: The process of defect movement in a line pattern.
